# Association of *ZNF608* Polymorphisms With House Dust Mite‐Induced Allergic Rhinitis

**DOI:** 10.1002/clt2.70081

**Published:** 2025-08-06

**Authors:** Huiqin Li, Fang Gao, Xuyan Liu, Haoran Shen, Mulong Du, Lei Cheng, Huihui Zhang, Zhengdong Zhang, Meiping Lu, Rui Zheng

**Affiliations:** ^1^ Departments of Environmental Genomics and Genetic Toxicology The Key Laboratory of Modern Toxicology of Ministry of Education Center for Global Health Jiangsu Key Laboratory of Cancer Biomarkers Prevention and Treatment Collaborative Innovation Center for Cancer Personalized Medicine School of Public Health Nanjing Medical University Nanjing China; ^2^ Institute of Clinical Research The Affiliated Taizhou People's Hospital of Nanjing Medical University Taizhou School of Clinical Medicine Nanjing Medical University Nanjing China; ^3^ Department of Urology The Third Affiliated Hospital of Nanjing Medical University (Changzhou Second People's Hospital) Changzhou China; ^4^ School of Pediatrics Nanjing Medical University Nanjing China; ^5^ Department of Biostatistics Center for Global Health School of Public Health Nanjing Medical University Nanjing China; ^6^ Department of Otorhinolaryngology & Clinical Allergy Center The First Affiliated Hospital Nanjing Medical University Nanjing China; ^7^ Department of Public Health Robbins College of Health and Human Sciences Baylor University Waco Texas USA; ^8^ Department of Occupational, Medicine and Environmental Health, School of Public Health Key Laboratory of Public Health Safety and Emergency Prevention and Control Technology of Higher Education Institutions in Jiangsu Province, Nanjing Medical University Nanjing China

**Keywords:** allergic rhinitis, genetic variants, immune cell, polygenic risk score, *ZNF608*

## Abstract

**Background:**

Genetic factors contribute essentially to the pathophysiology of house dust mite (HDM)‐induced allergic rhinitis. Previous studies mainly focused on the biological pathogenesis, but the heritability remains poorly explained.

**Methods:**

A genome‐wide gene association analysis (GWGAS) integrating joint‐genetic variant effects at the gene level was initially conducted on allergic rhinitis, validated by differential gene expression analysis. A weighted polygenic risk score (wPRS) was used to proxy the cumulative effect of candidate genetic variants in key genes. Gene‐set analysis and eQTL analysis were performed to explore the immunologic pathway and genetic regulation of the key gene.

**Results:**

*ZNF608* was identified as the key gene involving HDM‐induced allergic rhinitis risk (*p* = 1.23 × 10^−6^), which was highly expressed in nasal epithelium cells of allergic rhinitis patients (*p* = 0.041). Furthermore, a wPRS of five significant variants, rs6862252, rs10067299, rs10042766, rs6866116, and rs79679768 in the *ZNF608*, showed the cumulative effect was associated with the increased HDM‐induced allergic rhinitis risk (odds ratio [OR] = 1.40, 95% confidence interval [CI] = 1.18–1.65, *p* = 1.18 × 10^−4^), with varied effects under diverse conditions of nasal symptoms. Additionally, both rs6862252 G allele and rs10042766 T allele elevated the expression of *ZNF608* involving in state and perturbation of immune cells, such as B cell, T cell, and dendritic cell, contributing to HDM‐induced allergic rhinitis.

**Conclusion:**

This study highlights the key gene *ZNF608* of HDM‐induced allergic rhinitis, which may lay the groundwork for risk assessment and early diagnosis of allergic rhinitis.

**Abbreviations**
ATAC: Assay for Transposase‐Accessible Chromatin
CHB: Han Chinese in Beijing
CI: confidence interval
DCs: dendritic cells
*Der f*
: *Dermatophagoides farina*
*Der p*
: *Dermatophagoides pteronyssinus*
DN B: double negative B
eQTL: expression quantitative loci
GEO: Gene Expression Omnibus
GWAS: genome‐wide association study
GWGAS: genome‐wide gene association analysis
HDM: house dust mite
HWE: Hardy‐Weinberg equilibrium
JPT: Japanese in Tokyo
LD: linkage disequilibrium
LDG: low‐density granulocytes
MAF: minor allele frequency
MAGMA: Multi‐marker Analysis of GenoMic Annotation
mDC: myeloid dendritic cells
MFE: minimum free energy
MHC: major histocompatibility complex
Neu: neutrophils
OR: odds ratio
pDC: plasmacytoid dendritic cells
SM B: switched memory B
SNP: single nucleotide polymorphism
SYK: spleen tyrosine kinase
TNSS: total nasal symptom score
USM B: unswitched memory B
wPRS: weighted polygenic risk score

## 1 Introduction

Allergic rhinitis is a type of nasal mucosa inflammation to aeroallergens driven by immunologic hypersensitivity [1, 2]. The prevalence of allergic rhinitis varies across populations and has continued to increase, even up to 50% in some regions [3, 4]. Clinically, allergic rhinitis results in repeated sneezing, rhinorrhea, nasal itching, and nasal obstruction, leading to decreased life quality and increased healthcare costs [5, 6]. Various immune cells are recognized to play a crucial role in immunoglobulin E (IgE)‐mediated allergic response among allergic rhinitis, such as type 2 innate lymphocytes (ILC2s), T helper 2 (Th2) cells, and B cells [1, 7, 8]. House dust mite (HDM) is one of the primary indoor environmental allergens that impact the development and exacerbation of allergic rhinitis [9]. In China, HDM is responsible for most allergic rhinitis patients [10, 11], especially in South China [12]. However, the etiology of HDM‐induced allergic rhinitis remains elusive, and identifying key targets is pivotal to the diagnosis and management of allergic rhinitis induced by HDM.

The high heritability of allergic rhinitis indicates a crucial genetic contribution to the etiology [2, 13, 14]. Genome‐wide association study (GWAS) has been widely used for genetic susceptibility to multiple diseases, including HDM‐induced allergic rhinitis. Our groups and other teams have identified multiple single nucleotide polymorphisms (SNPs) associated with HDM‐induced allergic rhinitis [15]. We have reported that individuals with the AA genotype of rs754466 in the Hippo pathway gene *DLG5* showed a significantly decreased susceptibility to HDM‐induced allergic rhinitis [16]. Recently, our study has indicated that the rs2305128 and rs1868088 in two ferroptosis‐related genes, *ENPP2* and *EPAS1*, could increase the HDM‐induced allergic rhinitis risk, and a cumulative effect occurs with the increasing number of risk genotypes [17]. However, these efforts have been limited to specific biological pathways and could account for only a portion of heritability. Further study is required to elucidate the genetic component of HDM‐induced allergic rhinitis pathophysiology sufficiently.

Genome‐wide gene association study (GWGAS) enables the examination of joint‐SNP effects at the gene‐based level compared to typical GWAS testing on a single variant, which allows for the aggregation of multiple genetic evidence in a gene and the discovery of novel gene associations at whole genome level [18, 19]. It could help to facilitate the prioritization of candidate genes. In this study, we aimed to carry out a hypothesis‐free genome‐wide gene association study in a Chinese population to identify the key genes of HDM‐induced allergic rhinitis.

## 2 Methods

### 2.1 Study Population

A total of 222 cases of HDM‐induced allergic rhinitis were recruited from the First Affiliated Hospital of Nanjing Medical University from May 2008 to October 2017. The cases had a mean age of 23.23 years, with 128 (57.7%) participants being male. Allergic status was evaluated through serum allergen testing, including log transformed total IgE levels (mean ± SD: 2.09 ± 0.47), specific IgE to *Dermatophagoides pteronyssinus* (mean ± SD: 1.26 ± 0.59) and *Dermatophagoides farina* (mean ± SD: 0.97 ± 0.63). Total nasal symptom score (TNSS), evaluated by clinical symptoms, including sneezing, rhinorrhea, nasal itching, and nasal obstruction, were also available in patients (Supporting Information S1: Table S1). With age and sex‐matched, 237 healthy controls were randomly drawn from physical examination centers in the same geographical region. Written informed consent was obtained from all the individuals. More descriptions and characteristics were detailed previously [16, 17].

### 2.2 Genotyping and Quality Control

The genotyping procedures could be found in the previous study [16]. SNPs located in the major histocompatibility complex (MHC) region were excluded for its complexity in linkage disequilibrium (LD) structure. Details of individual genotyping data, including the imputation, of all samples were previously published [16]. Ancestry was examined using principal components analysis on the genotype data. A total of 3,050,608 autosomal SNPs were available.

### 2.3 Genome‐Wide Gene‐Based Analysis (GWGAS)

Genome‐wide gene association analysis (GWGAS) allows for the aggregation of associations from multiple SNPs annotated to each gene [20, 21]. A GWGAS that encompassed all 19,427 protein‐coding genes was implemented by Multi‐marker Analysis of GenoMic Annotation (MAGMA) v1.10 [22]. Individual‐level sequence data was utilized as input for the analysis. A total of 1,245,443 SNPs were then mapped into 16,408 genes for further analyses in this study. A stringent Bonferroni correction was applied to control the multiple comparisons and the genome‐wide significance threshold was defined at *p* < 3.05 × 10^−6^ (0.05/16,408). The principal components regression model at default settings was employed for GWGAS, with age, gender, and the top three genetic principal components added as covariates.

### 2.4 Publicly Available Database

Gene expression profiling of nasal epithelial cells from a cross‐sectional study (GSE44037), comprising five allergic rhinitis and six healthy controls [23], was downloaded from the Gene Expression Omnibus (GEO) database. Besides, the ImmuNexUT [24] database was applied to assess the expression pattern of key genes and to identify the expression quantitative loci (eQTL) regulatory effects for candidate SNPs on genes in multiple immune cell types from the Japanese population (https://www.immunexut.org/).

### 2.5 Immune‐Related Gene‐Set Analysis

A competitive gene‐set analysis modeling as a linear regression was implemented using MAGMA with default parameters. The gene *p* values and gene‐level data matrix were supplied from GWGAS. The pathways, in the immunologic signature gene set database [25] from MSigDB, harboring *ZNF608* were manually assigned to further analysis, which represents cell states and perturbations within the immune system. A total of 64 eligible gene sets were included in the present investigation. The competitive gene set *p*‐values were calculated on the GWGAS to evaluate the association of immune‐related gene sets with HDM‐induced allergic rhinitis.

### 2.6 Selection of SNPs

SNPs located in the key genes were extracted from subjects of Han Chinese in Beijing (CHB) and Japanese in Tokyo (JPT) from the 1000 Genomes Project. The quality control was processed as the criteria: (a) call rate > 95%, (b) MAF > 0.05, and (c) Hardy‐Weinberg equilibrium (HWE) *p* value > 1 × 10^−6^. To screen out the independent variants, pairwise linkage disequilibrium (LD) analysis was performed, and *r* ^2^ = 0.80 was set as a cutoff. Then, the effects of candidate SNPs on HDM‐induced allergic rhinitis were determined by a logistic regression model, with age and sex adjusted. The top three principal components were also utilized as covariates to control for population stratification.

### 2.7 Weighted Polygenic Risk Score (wPRS) Associated With Allergic Rhinitis

A weighted polygenic risk score (wPRS) was generated as an alternative strategy to assess the cumulative effect of five susceptibility loci. The wPRS of each individual was calculated as the sum of the risk alleles weighted by their effect sizes following the formula, wPRS = $\sum_{i=1}^{n}\beta_{i}{\text{SNP}}_{i}$, where *n* denotes the number of SNPs, $\beta_{i}$ is the coefficient from the above additive model, and ${\text{SNP}}_{i}$ is the risk allele number (coded by 0, 1, or 2).

### 2.8 Genetic Function Annotation

RegulomeDB [26, 27] v2.2 (https://regulomedb.org/), HaploReg [28] v4.2 (https://pubs.broadinstitute.org/mammals/haploreg/haploreg.php), and VARAdb [29] (https://bio.liclab.net/VARAdb/search.php) were queried to obtain potential regulatory functionality and annotation of genetic variants located in key gene. 3DSNP v2.0 (https://omic.tech/3dsnpv2/) was applied to explore and visualize their 3D chromatin structure and interaction [30]. Besides, RNAfold WebServer (http://rna.tbi.univie.ac.at/cgi‐bin/RNAWebSuite/RNAfold.cgi) was used to predict secondary structures of given RNA sequences of SNPs [31, 32].

### 2.9 Statistical Analysis

The logistic model was applied to calculate the odds ratio (OR) and 95% confidence intervals (CIs) for the effect of SNPs in *ZNF608* on the risk of allergic rhinitis. Differential gene expression analysis between two groups was guided by the *limma* pipeline in R from GEO2R [33]. *p* value < 0.05 was considered statistically significant unless otherwise defined. All statistical analyses were conducted by R 4.0.5 and PLINK v1. 90.

## 3 Results

### 3.1 Study Design

**FIGURE 1:**
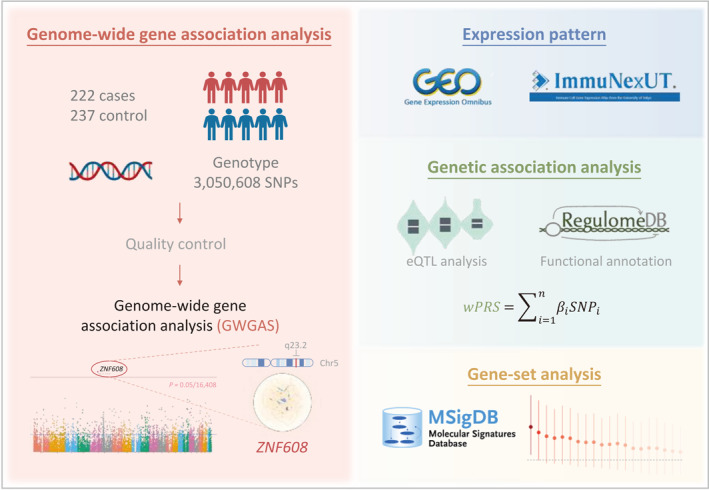
Flow chart of the study. The left pink part represents the identification of the key gene *ZNF608* associated with HDM‐induced allergic rhinitis risk by a genome‐wide gene association analysis. Then, we explored the expression pattern of *ZNF608* between patients with allergic rhinitis and controls. To further investigate the genetic predisposition to allergic rhinitis, we performed genetic association studies to identify candidate SNPs. The eQTL analysis and functional annotation were applied to characterize the potential biological properties. A weighted polygenic risk score was calculated to assess the joint effect. Moreover, pathway enrichment analysis was conducted based on immunological signature gene sets harboring *ZNF608*.

The flowchart of this study is depicted in Figure 1. Briefly, a GWGAS was applied to HDM‐induced allergic rhinitis phenotype to identify the key gene, for which their expressions were then validated in nasal epithelium cells between the allergic rhinitis cases and controls, as well as in immune cell types. Additionally, genetic association analysis was performed on each SNP in the key genes, and a wPRS was calculated to assess joint effects on allergic rhinitis. *In silico* function annotation was carried out to explore potential biological functionality. eQTL analysis was then performed on candidate SNPs that might affect gene expression and thereby participate in HDM‐induced allergic rhinitis susceptibility. Furthermore, immune‐related gene‐set analysis harboring the key gene was conducted to elucidate the biological pathway the key gene might regulate in HDM‐induced allergic rhinitis risk.

### 3.2 Identification of Key Gene *ZNF608* on Allergic Rhinitis Risk

**FIGURE 2:**
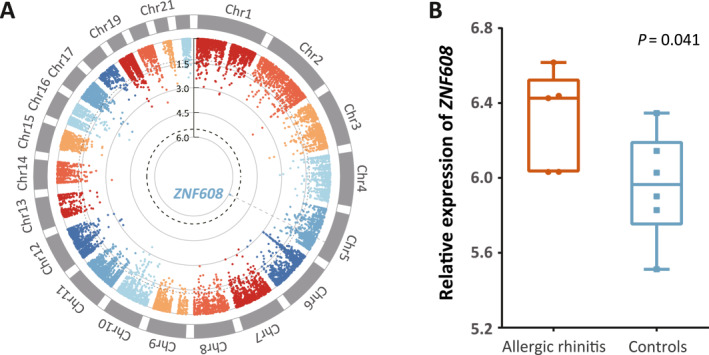
*ZNF60*8 is associated with risk of HDM‐induced allergic rhinitis. (A) Circle Manhattan plot depicts the results of genome‐wide gene association analysis by MAGMA. The black dotted line represents genome‐level significant level (*p* = 3.05 × 10^−6^) and the gray one represents significant level (*p* = 0.05). (B) The expression pattern of *ZNF608* in nasal epithelium cells between five patients diagnosed with allergic rhinitis and six controls from GSE44037. Individual data points are overlaid to show sample distribution.

We first performed a genome‐wide gene association study to identify the key genes related to HDM‐induced allergic rhinitis risk and found that *ZNF608* was the most significant and survived at genome‐wide significance (Z score = 4.71, *p* = 1.23 × 10^−6^, Figure 2A and Supporting Information S1: Table S2), indicating that *ZNF608* is closely related to HDM‐induced allergic rhinitis. Subsequently, we bioinformatically analyzed the transcriptome of nasal epithelial cells and observed that *ZNF608* was upregulated in patients with allergic rhinitis than that in controls, consistent with the result from GWGAS (*p* = 0.041, Figure 2B). While no significant association were found in European population, indicating its specific role in Asia population (Supporting Information S1: Table S3). These results suggested that *ZNF608* plays an important role in HDM‐induced allergic rhinitis.

### 3.3 Genetic Association Between *ZNF608* Genetic Variants and Allergic Rhinitis Susceptibility

**FIGURE 3:**
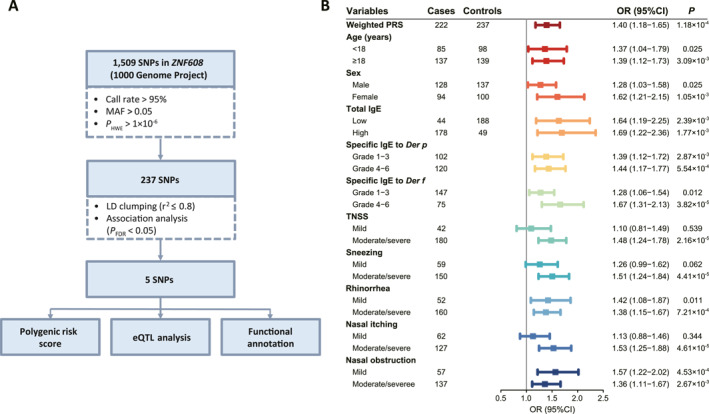
Identification of genetic variants in *ZNF608* and their cumulative effects to HDM‐induced allergic rhinitis susceptibility. (A) Selection of variants in *ZNF608* involved in HDM‐induced allergic rhinitis. After excluding SNPs showed high linkage disequilibrium (*r* ^2^ > 0.8), 237 SNPs were retained for further logistic analysis. Five SNPs in *ZNF608* were significantly associated with allergic rhinitis. LD, linkage disequilibrium. FDR, false discovery rate. (B) Forest plot illustrates the cumulative effects and stratification analysis of the association between wPRS constructed by five associated SNPs in *ZNF608* and the susceptibility to HDM‐induced allergic rhinitis. p, *p* value adjusted for age, sex and the first three PCs. TNSS, total nasal symptom score. There is missing information on sneezing, rhinorrhea, nasal itching, and nasal obstruction for some patients.

Considering the importance of genetic factors in HDM‐induced allergic rhinitis, we further aimed to identify potentially functional SNPs in the *ZNF608* for deep exploration of the genetic etiology of allergic rhinitis. As shown in Figure 3A and Table 1, a total of 1509 SNPs were obtained from *ZNF608* and 237 SNPs were retained after quality control. Notably, five independent SNPs were observed to be implicated by HDM‐induced allergic rhinitis under an additive model after the LD clumping procedure (*P*_FDR_ < 0.05). Among these, three variants exhibited a prominent risk effect, including rs6862252 T > G (OR = 1.91, 95% CI = 1.36–2.67, *P*_FDR_ = 2.75 × 10^−3^), rs10042766 C > T (OR = 1.60, 95% CI = 1.17–2.17, *P*_FDR_ = 0.016) and rs79679768 A > G (OR = 1.67, 95% CI = 1.12–2.47, *P*_FDR_ = 0.037). Besides, rs10067299 G > A (OR = 0.66, 95% CI = 0.50–0.86, *P*_FDR_ = 0.016) and rs6866116 T > C (OR = 0.70, 95% CI = 0.54–0.91, *P*_FDR_ = 0.032) are identified to be protective against allergic rhinitis. These findings indicate that the dominant roles of these five SNPs in *ZNF608* are involved in genetic predisposition to HDM‐induced allergic rhinitis.

**TABLE 1:** Association between 16 SNPs in *ZNF608* after LD clumping and HDM‐induced allergic rhinitis risk.

SNP	Chr	BP	A1^a^	A2^b^	EAF (case/control)	OR (95% CI)	*p* ^c^	*P*_FDR_
**rs6862252**	5	123,983,915	G	T	0.291/0.187	1.91 (1.36–2.67)	1.62 × 10^−4^	**2.75** × **10** ^**−** **3**^
**rs10067299**	5	123,996,609	A	G	0.340/0.441	0.66 (0.50–0.86)	2.24 × 10^−3^	**0.016**
**rs10042766**	5	123,989,154	T	C	0.310/0.224	1.60 (1.17–2.17)	2.87 × 10^−3^	**0.016**
**rs6866116**	5	123,980,872	C	T	0.394/0.483	0.70 (0.54–0.91)	7.63 × 10^−3^	**0.032**
**rs79679768**	5	124,034,164	G	A	0.173/0.118	1.67 (1.12–2.47)	0.011	**0.037**
rs7705693	5	124,084,365	T	C	0.223/0.173	1.38 (0.99–1.90)	0.057	0.162
rs12513661	5	124,038,251	A	G	0.137/0.107	1.38 (0.91–2.09)	0.130	0.283
rs79399452	5	124,062,907	C	T	0.094/0.129	0.72 (0.47–1.10)	0.133	0.283
rs4836111	5	124,042,644	G	A	0.187/0.160	1.24 (0.86–1.77)	0.251	0.449
rs13179054	5	124,000,865	T	C	0.264/0.231	1.19 (0.87–1.61)	0.273	0.449
rs4579258	5	124,037,371	A	G	0.388/0.355	1.16 (0.88–1.54)	0.291	0.449
rs35881280	5	124,043,382	T	C	0.122/0.144	0.84 (0.57–1.24)	0.380	0.538
rs13163827	5	124,082,553	A	G	0.093/0.113	0.84 (0.54–1.30)	0.426	0.557
rs3860117	5	124,043,018	A	G	0.259/0.245	1.11 (0.82–1.49)	0.516	0.626
rs4836112	5	124,043,721	T	C	0.186/0.202	0.92 (0.65–1.29)	0.610	0.691
rs11241764	5	124,044,428	T	C	0.061/0.057	1.06 (0.60–1.85)	0.848	0.884

*Note:* The bold values indicate significant SNPs with their corresponding *p* values.Abbreviations: Chr, chromosome; CI, confidence interval; EAF, effect allele frequency; FDR, false discovery rate; OR, odds ratio.

^a^ Effect allele.

^b^ Reference allele.

^c^ Age, sex and the top three principal components were adjusted in the additive model.

To investigate the cumulative effects of SNPs in the key gene *ZNF608*, we constructed a wPRS as a proxy and conducted a stratified analysis based on demographic and clinical characteristics (Supporting Information S1: Figure S1). As presented in Figure 3B and Table 2, the wPRS showed a notable combined risk effect of SNPs located in *ZNF608* on susceptibility to HDM‐induced allergic rhinitis (OR = 1.40, 95% CI = 1.18–1.65, *p* = 1.18 × 10^−4^). Stratification analysis of allergic rhinitis‐related phenotypes revealed a significant association of wPRS with an increased risk of HDM‐induced allergic rhinitis in patients experiencing moderate/severe TNSS, sneezing, and nasal itching, with a less pronounced effect observed in individuals with mild symptoms (Figure 3B). These findings indicated that SNPs in *ZNF608* have a joint effect on the risk of HDM‐induced allergic rhinitis, which varies among individuals with different severity of nasal symptoms and specific IgE to *Der f*.

**TABLE 2:** Joint effect and stratification analysis of wPRS based on five susceptibility SNPs in *ZNF608* by with the risk of allergic rhinitis.

Variables	Cases (*n* = 222) *N* (%)	Controls (*n* = 237) *N* (%)	OR (95% CI)	*p* ^a^	*p* ^*^
wPRS			1.40 (1.18–1.65)	1.18 × 10^−4^	
Age (years)					0.914
< 18	85 (38.3)	98 (41.4)	1.37 (1.04–1.79)	0.025	
≥ 18	137 (61.7)	139 (58.6)	1.39 (1.12–1.73)	3.09 × 10^−3^	
Sex					0.248
Male	128 (57.7)	137 (57.8)	1.28 (1.03–1.58)	0.025	
Female	94 (42.3)	100 (42.2)	1.62 (1.21–2.15)	1.05 × 10^−3^	
Total IgE^b^					0.871
Low	44 (19.8)	188 (79.3)	1.64 (1.19–2.25)	2.39 × 10^−3^	
High	178 (80.2)	49 (20.7)	1.69 (1.22–2.36)	1.77 × 10^−3^	

Abbreviations: CI, confidence interval; OR, odds ratio; wPRS, weighted polygenic risk score.

^a^ Adjusted for age, sex, and the top three principal components in logistic regression model.

^b^ Cut‐off value of total IgE = 60.45 kU/L, low level of total IgE < 60.45 kU/L, high level of total IgE ≥ 60.45 kU/L.

^*^ *p* value for interaction analysis.

### 3.4 Functional Characterization of Candidate SNPs and Genetic Regulation With *ZNF608* in Immune Cells

**FIGURE 4:**
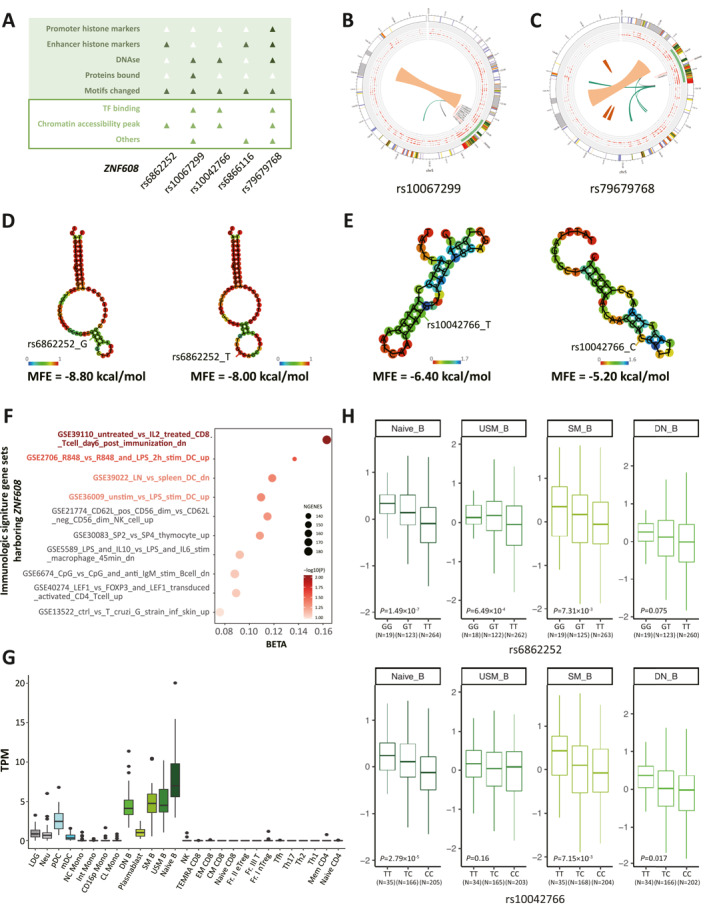
The biological function and eQTL effects of SNPs in *ZNF608*. (A) Functional annotation of five SNPs based on HaploReg (upon filled in green) and RegulomeDB (bottom filled in white). (B–C) Circos plots of chromatin loops, states and 3D interaction of rs10067299 and rs79679768 in *ZNF608* using 3DSNP. (D–E) MFE structure plots of rs6862252 and rs10042766 colored by base‐pair probabilities based on SNP‐fold website. (F) Gene‐set analysis of immune signature gene‐set harboring *ZNF608* and HDM‐induced allergic rhinitis. The size of point represents the genes involved in the pathway. The range of color represents the significance. (G) The expression level of *ZNF608* in 28 immune cell types from ImmuNexUT. (H) Box plots depict the eQTL effects between rs6862252 and rs10042766 genotypes and *ZNF608* expression of 423 donors from ImmuNexUT. The number of samples for each genotype is shown below *X*‐axis.

We next conducted in silico functional annotation analysis to explore the potential functions of these candidate SNPs in *ZNF608*. HaploReg and RegulomeDB revealed that these variants are likely to be functionally active for significant enrichment of chromatin accessibility peak, altered sequence motifs, and DNAse hypersensitive sites, suggesting potential regulatory effects on *ZNF608* (Figure 4A). Additionally, we observed these five SNPs were located in enhancer regions in multiple cell type colocalized with histone markers and Assay for Transposase‐Accessible Chromatin (ATAC) peaks for rs10067299 and rs79679768 (Figure 4B,C, Supporting Information S1: Figure S2 and Table S4). Functional annotation provided evidence of 15 transcription factor binding sites in several cell types for rs10067299, and transcription factor *FOXA1* in one cell type for rs79679768 (Supporting Information S1: Table S5). Furthermore, we observed that the RNA secondary structure and minimum free energy (MFE) were predicted to be altered by rs6862252 T > G and rs10042766 C > T based on RNAfold (Figure 4D,E and Supporting Information S1: Figure S3). These findings suggested that *ZNF608* genetic variants are potential regulatory variants and may regulate chromatin accessibility, influence transcription factor binding, and interfere the RNA secondary structure to contribute to the susceptibility of allergic rhinitis.

To elucidate the biological pathway regulated by *ZNF608* in HDM‐induced allergic rhinitis, we further conducted a competitive gene‐set analysis by MAGMA. Since allergic rhinitis was convinced to be an immune‐related disease, we primarily focused on immunologic signature gene sets and included 64 pathways harboring *ZNF608* manually. The competitive gene set analysis resulted in four immune‐related gene sets that displayed associations with HDM‐induced allergic rhinitis (Figure 4F and Supporting Information S1: Table S6). Among these, the most significant pathway showed that *ZNF608* played a crucial role in the rapid and substantial development of antigen‐specific CD8^+^ T memory cells (*p* = 8.95 × 10^−3^, Figure 4F and Supporting Information S1: Table S6). These enriched gene sets indicated that *ZNF608* might play an important role in the state and perturbation of immune cells involving allergic rhinitis.

To further explore the role and mechanism of *ZNF608* in immune cells based on the results of gene‐sets enrichment analysis mentioned above, we assessed the expression pattern of *ZNF608* and performed eQTL analysis, *ZNF608* enriched in nine types of immune cells, including low‐density granulocytes (LDG), neutrophils (Neu), plasmacytoid dendritic cells (pDC), myeloid dendritic cells (mDC), double negative B (DN B) cells, plasma blast, switched memory B (SM B) cells, unswitched memory B(USM B) cells, and Naïve B cells, which most highly expressed in Naïve B cells (Figure 4G). Intriguingly, eQTL analysis showed that both rs6862252 T > G and rs10042766 C > T could upregulate the expression of *ZNF608* in naïve B cells (*P*_rs6862252_ = 1.49 × 10^−7^ and *P*_rs10042766_ = 2.79 × 10^−5^) and SM B cell (*P*_rs6862252_ = 7.31 × 10^−3^ and *P*_rs10042766_ = 7.15 × 10^−3^). Moreover, rs6268252 T > G exhibited a positive correlation with *ZNF608* expression in USM B cell (*p* = 6.49 × 10^−4^) and rs10042766 C > T in DN B cell (*p* = 0.017) (Figure 4H). These results indicated that rs6268252 T > G and rs10042766 C > T could upregulate the expression of *ZNF608* in the immune cells involved in HDM‐induced allergic rhinitis.

## 4 Discussion

Allergic rhinitis is a common chronic respiratory condition resulting from a hyperresponse of the immune system with an increasing tendency of prevalence [34, 35]. Previous studies have found that HDM sensitization is major for the occurrence of allergic rhinitis [36, 37]. The prevalence of HDM‐induced allergic rhinitis could be ascribed partly to a predisposed genetic background [38]. GWAS have revealed numerous associated SNPs [2], however, most of these associations are confined to specific biological pathways or functional genes. Recently, GWGAS has emerged as a method to identify novel signals at the gene level and facilitate the prioritization of candidate genes [21]. Compared to traditional GWAS analysis, GWGAS allows for the aggregation of associations from multiple SNPs annotated to each gene [20, 21]. For instance, Jansen et al. identified 97 genes associated with Alzheimer's disease under the GWGAS analytical framework [20]. However, few studies have focused on HDM‐induced allergic rhinitis through GWGAS until now. In this study, we first performed a GWGAS study based on genomic data and identified the key gene *ZNF608* associated with the susceptibility to HDM‐induced allergic rhinitis in Chinese individuals, validated by differential mRNA expression analysis based on transcriptome data.

*ZNF608* (zinc finger protein 608), a protein‐coding gene, is ubiquitously expressed in lung tissue [39]. Previous studies have shown that male‐specific polymorphisms in *ZNF608* are associated with asthma‐COPD phenotype [40]. Liu et al. [41] demonstrated that *ZNF608* may be a potential target in both short‐ and long‐term SARS‐CoV/SARS‐CoV‐2 infection under the ferret lung model, which features analogously in symptom and pathology with humans. These findings suggested the crucial role of *ZNF608* in respiratory diseases. Besides, transcription factor *ZNF608* exhibits an inverse expression pattern with spleen tyrosine kinase (SYK), which is essential in IgE‐mediated immune response, unique in CD34B cells [42]. These suggested that *ZNF608* is an immune‐related gene. In our study, we found that *ZNF608* is expressed in immune cells, especially in Naïve B cells. While our study demonstrates a statistically significant association between *ZNF608* and allergic rhinitis, the sample size may limit detection of weaker genetic effects. Independent replication in larger cohorts, combined with mechanistic studies, will be essential to validate these findings and elucidate *ZNF608*'s role in allergic rhinitis.

We next conducted a gene‐set analysis focused on immune‐related pathways harboring key gene *ZNF608* on HDM‐induced allergic rhinitis in this study, which highlighted the immunoregulatory role of *ZNF608* in immune cells contributing to allergic rhinitis, such as T cell and dendritic cells (DCs). Allergic rhinitis is a well‐known chronic inflammatory disease of the nasal mucosa mediated by IgE [43]. T cell subsets, B cells, and DCs were recognized to contribute to allergic rhinitis pathogenesis by noticeable publications [8, 44]. It was reported that the percentages of B cells including naïve B cells in peripheral blood are characteristics of allergic rhinitis patients [45]. A previous study has shown that CD8^+^ Tregs could ameliorate the inflammatory condition in allergic rhinitis [46]. In addition, Ogulur et al. comprehensively reviewed and highlighted the crucial role of immune cells and the molecules in the pathogenesis of allergic rhinitis and exemplified that the expression of CD23 on B cells has been identified as a biomarker of allergic rhinitis and serves as the bridge between T‐B cell interaction in allergic rhinitis [4, 47], suggesting a role of *ZNF608* for T‐B cell interaction in HDM‐induced allergic rhinitis. Moreover, human allergen‐DNA‐transfected DCs were verified to stimulate CD4 and CD8 T cells and had the potential for treatment of IgE‐mediated allergic diseases [48]. These findings supported the regulatory mechanisms of *ZNF608* in the immune cell perturbations within the immune system associated with HDM‐induced allergic rhinitis, including B cells, T cells, and DCs, and their interaction. However, biological experiments for detailed mechanisms in immune cells were not further investigated in our study, which might help to elucidate the immune pathogenesis of *ZNF608* in allergic rhinitis.

GWAS could help to elucidate the genetic susceptibility, and it has identified several loci associated with allergic rhinitis [49, 50, 51], many of which are implicated in regulatory effects on the immune system. Significant HLA alleles in allergic rhinitis play diverse roles in IgE‐related immune mechanisms [2]. Moreover, *MRPL4* at 19q13 is related to the inflammatory adhesion process, and *C11orf30–LRRC32* at 11q13 is associated with immune tolerance [1, 52]. In our study, we further performed a logistic regression analysis and identified five associations between SNPs in *ZNF608* and susceptibility to HDM‐induced allergic rhinitis. A polygenic risk score is an effective method to assess the joint effect of candidate SNPs [53]. Therefore, to further investigate the role of the cumulation effect of *ZNF608* genetic variants on allergic rhinitis, we constructed a wPRS and our results indicated that wPRS showed a 1.40‐fold per standard deviation (SD) increase in risk of HDM‐induced allergic rhinitis. Furthermore, stratification analysis suggested the joint effect differed in individuals who suffered mild or severe nasal symptoms. A higher risk in patients with severe TNSS compared to those with mild symptoms. Evidence has shown that the frequencies of switched memory B cells and CD23 expression on switched memory B cells are both positively associated with symptom scores in allergic rhinitis patients [47]. The decrease of allergen‐specific IL‐4^+^ T_FH_2 cells and the increase in T_FR_ cells in HDM‐induced allergic rhinitis are related to the improvement of symptoms [54, 55]. These indicated that five SNPs might modulate the expression of *ZNF608*, affecting immune cell states and perturbations in the immune system thus increasing the risk of allergic rhinitis and the occurrence of nasal symptoms. Moreover, it is well‐recognized that the environment has a substantial impact on HDM‐induced allergic rhinitis [56]. Nevertheless, a lack of related information resulted in a blank in the role of environmental factors in the association and the gene‐environmental interaction.

Considering the involvement of *ZNF608* in immune, we assessed the eQTL effect of the above five SNPs in multiple immune cells and found that the G allele of rs6862252 and T allele of rs10042766 could genetically up‐regulate the expression of *ZNF608* in B cells, including naïve B cells, SM B cells. It indicated that *ZNF608* might be regulated by rs6862252 and rs10042766 within the immune microenvironment to contribute to allergic rhinitis. Although no significant eQTL signals were observed in immune cells for rs10067299, rs6866116 and rs79679768, they may modulate *ZNF608* through other pathways to participate in susceptibility to allergic rhinitis. The lack of transcriptome data in the nasal mucosa has led to a gap in variants and *ZNF608* expression.

In summary, we conducted a genome‐wide gene association analysis and reported the genetic variants of key gene *ZNF608* associated with risk of HDM‐induced allergic rhinitis. eQTL analysis and gene set analysis demonstrated the involvement of genetic regulation between the rs6862252 and rs10042766 with *ZNF608* in immune cells, for example naïve B cells, SM B cells. Our findings lay the groundwork for further functional evaluations of genetic biomarkers implicated by HDM‐induced allergic rhinitis and provide novel insights into risk assessment and early diagnosis. Our results also broaden the understanding of the immune‐related pathogenesis of HDM‐induced allergic rhinitis.

## Author Contributions

**Huiqin Li:** visualization, formal analysis, writing – original draft. **Fang Gao:** formal analysis, writing – original draft. **Xuyan Liu:** validation. **Haoran Shen:** validation. **Mulong Du:** conceptualization, writing – review and editing. **Lei Cheng:** funding acquisition, conceptualization, writing – review and editing. **Huihui Zhang:** writing – review and editing. **Zhengdong Zhang:** writing – review and editing, conceptualization. **Meiping Lu:** writing – review and editing, funding acquisition, conceptualization. **Rui Zheng:** writing – review and editing, conceptualization.

## Ethics Statement

All participants signed informed consent forms. The project was approved by the Institutional Review Boards of The First Affiliated Hospital with Nanjing Medical University (2024‐SRFA‐085).

## Consent

The authors have nothing to report.

## Conflicts of Interest

The authors declare no conflicts of interest.

## Supporting information

Supporting Information S1

## Data Availability

Data in this study are available upon request to the corresponding author.
