# Supplementary material for: Association of ZNF608 Polymorphisms With House Dust Mite‐Induced Allergic Rhinitis
Source: Clin Transl Allergy. 2025 Aug 6;15(8):e70081. doi: 10.1002/clt2.70081 (PMC12326142; doi:10.1002/clt2.70081)

**Supplementary Tables**

**Table S1.** The clinical characteristics of patients with allergic rhinitis involved in the study.

**Table S2.** Results of top 20 genes associated with allergic rhinitis based on genome-wide gene association study by MAGMA.

**Table S3.** The results of gene analysis of *ZNF608* and allergic rhinitis from UK biobank and FinnGen by MAGMA.

**Table S4.** Functional annotation of five SNPs in *ZNF608* using VARAdb.

**Table S5.** Functional annotation of five SNPs in *ZNF608* using 3DSNP.

**Table S6**. Description of ten immune-related gene sets from MSigDB (related to Fig. 4F).

**Supplementary Figures**

**Fig S1.** The distribution of weighted polygenic risk score (wPRS) based on 5 SNPs in *ZNF608*.

**Fig S2.** Visualization of chromatin loops, states and 3D interaction of the SNPs in *ZNF608* using 3DSNP.

**Fig S3.** The change of minimum free energy caused by rs6862252 T>G and rs10042766 C>T based on RNAfold website.

**Table S1.** The clinical characteristics of patients with allergic rhinitis involved in the study.

| **Variables** | **Value** | **Description** |
| --- | --- | --- |
| **Age** | 23.23 ± 12.18 | Mean ± SD. |
| **Sex** |  | N (%). |
| Male | 128 (57.7) |  |
| Female | 94 (42.3) |  |
| **Total IgE** | 2.09 ± 0.47 | Mean ± SD.  log kU/L. The levels of total IgE were log transformed to normalize the distribution. |
| **Total IgE** |  | N (%). |
| Low | 44 (19.8) | Total IgE < 60.45 kU/L. |
| High | 178 (80.2) | Total IgE ≥ 60.45 kU/L. |
| **Specific IgE to *Der p*** | 1.26 ± 0.59 | Mean ± SD.  log kU_A_/L. The levels of specific IgE (sIgE) to *Dermatophagoides pteronyssinus* were log transformed. |
| **Specific IgE to *Der p*** |  | N (%). |
| Grade 1–3 | 102 (45.9) | Grade 1: sIgE = 0.35-0.69 kU_A_/L, Grade 2: sIgE = 0.70-3.49 kU_A_/L, Grade 3: sIgE = 3.50-17.49 kU_A_/L. |
| Grade 4–6 | 120 (54.1) | Grade 4: sIgE = 17.50-49.99 kU_A_/L, Grade 5: sIgE = 50.0-100 kU_A_/L, Grade 6: sIgE > 100 kU_A_/L. |
| **Specific IgE to *Der f*** | 0.97 ± 0.63 | Mean ± SD.  log kU_A_/L. The levels of specific IgE to *Dermatophagoides* *farina* were log transformed. |
| **Specific IgE to *Der f*** |  | N (%). |
| Grade 1–3 | 147 (66.2) | Grade 1: sIgE = 0.35-0.69 kU_A_/L, Grade 2: sIgE = 0.70-3.49 kU_A_/L, Grade 3: sIgE = 3.50-17.49 kU_A_/L. |
| Grade 4–6 | 75 (33.8) | Grade 4: sIgE = 17.50-49.99 kU_A_/L, Grade 5: sIgE = 50.0-100 kU_A_/L, Grade 6: sIgE > 100 kU_A_/L. |
| **TNSS** |  | N (%).  On a scale of 0-12. TNSS, total nasal symptom score, was an indicator to comprehensively evaluate the severity of disease based on sneezing, rhinorrhea, nasal itching and nasal obstruction. The score of each symptom is: 0 = no symptoms; 1 = mild symptoms; 2 = moderate symptoms, and 3 = severe symptoms. |
| Mild | 42 (18.9) | Patients with TNSS ≤ 4. |
| Moderate/severe | 180 (81.1) | Patients with TNSS > 4. |
| **Sneezing** |  | N (%). |
| Mild | 59 (26.6) | Symptom clearly present, but easily tolerated. |
| Moderate/severe | 150 (67.6) | Definite awareness of symptom. |
| Missing | 13 (5.8) | Symptom causes interference with activities of daily living and/or sleeping. |
| **Rhinorrhea** |  | N (%). |
| Mild | 52 (23.4) |  |
| Moderate/severe | 160 (72.1) |  |
| Missing | 10 (4.5) |  |
| **Nasal itching** |  | N (%). |
| Mild | 62 (27.9) |  |
| Moderate/severe | 127 (57.2) |  |
| Missing | 33 (14.9) |  |
| **Nasal obstruction** |  | N (%). |
| Mild | 57 (25.7) |  |
| Moderate/severe | 137 (61.7) |  |
| Missing | 28 (12.6) |  |

**Table S2.** Results of top 20 genes associated with HDM-induced allergic rhinitis based on genome-wide gene association study by MAGMA.

| **Gene symbol** | **Chr** |  | **Start** | **Stop** | **N_SNPs_** | **Z-score** | ***P*^a^** |
| --- | --- | --- | --- | --- | --- | --- | --- |
| ***ZNF608*** | 5 |  | 123972606 | 124084517 | 124 | 4.71 | **1.23×10^-6^** |
| *DTYMK* | 2 |  | 242615157 | 242626383 | 42 | 3.70 | 1.09×10^-4^ |
| *KIAA0895L* | 16 |  | 67209505 | 67217883 | 1 | 3.63 | 1.39×10^-4^ |
| *SCO1* | 17 |  | 10583649 | 10600885 | 15 | 3.57 | 1.80×10^-4^ |
| *RBPJL* | 20 |  | 43935483 | 43946464 | 12 | 3.49 | 2.39×10^-4^ |
| *DNAJC19* | 3 |  | 180701497 | 180707562 | 2 | 3.49 | 2.44×10^-4^ |
| *COMP* | 19 |  | 18893583 | 18902114 | 5 | 3.37 | 3.76×10^-4^ |
| *PDZD7* | 10 |  | 102767440 | 102790914 | 2 | 3.36 | 3.96×10^-4^ |
| *CDH24* | 14 |  | 23516270 | 23526747 | 4 | 3.33 | 4.30×10^-4^ |
| *FXR1* | 3 |  | 180630096 | 180700541 | 73 | 3.31 | 4.74×10^-4^ |
| *SMARCC2* | 12 |  | 56555636 | 56583351 | 1 | 3.27 | 5.47×10^-4^ |
| *ING5* | 2 |  | 242641456 | 242668896 | 33 | 3.26 | 5.62×10^-4^ |
| *LHX1* | 17 |  | 35294772 | 35301915 | 8 | 3.24 | 5.97×10^-4^ |
| *CNGB1* | 16 |  | 57916244 | 58005020 | 79 | 3.23 | 6.21×10^-4^ |
| *NPVF* | 7 |  | 25264189 | 25268105 | 11 | 3.21 | 6.59×10^-4^ |
| *SOGA3* | 6 |  | 127759551 | 127841938 | 107 | 3.21 | 6.70×10^-4^ |
| *TSACC* | 1 |  | 156307105 | 156316786 | 7 | 3.20 | 6.86×10^-4^ |
| *PLCZ1* | 12 |  | 18836110 | 18891020 | 40 | 3.20 | 6.94×10^-4^ |
| *HOXC13* | 12 |  | 54332576 | 54340328 | 2 | 3.19 | 6.99×10^-4^ |
| *LRRC36* | 16 |  | 67360747 | 67419109 | 3 | 3.19 | 7.15×10^-4^ |

^a^Age, sex and the top three principal components were adjusted.

**Table S3.** The results of gene analysis of *ZNF608* and allergic rhinitis from UK biobank and FinnGen by MAGMA.

| **Gene symbol** | **Data source** | **N_cases_** | **N_controls_** | **N_SNPs_** | **Z-score** | ***P*^a^** |
| --- | --- | --- | --- | --- | --- | --- |
| *ZNF608* | UK Biobank | 1,531 | 398,757 | 226 | 0.04 | 0.485 |
| *ZNF608* | FinnGen | 15,569 | 474,650 | 233 | 0.57 | 0.283 |

*The GWAS summary statistics of allergic rhinitis was downloaded from database Pan-UK Biobank (<https://pan.ukbb.broadinstitute.org/>) and FinnGen R12 (https://r12.finngen.fi/).

**Table S4.** Functional annotation of five SNPs in *ZNF608* using VARAdb.

| **SNP** | **Chr** | **Position** | **Allele** | **Score** | **N_enhancer** | **N_ATAC** | **Risk_SNP** | **N_eQTL** | **N_disease** | **Variation_type** |
| --- | --- | --- | --- | --- | --- | --- | --- | --- | --- | --- |
| rs10067299 | 5 | 123996609 | G>A | 4 | 363 | 7 | No | 1 | / | Non-coding variation |
| rs6862252 | 5 | 123983915 | T>G | 4 | 94 | / | Yes | / | 2+ | Coding region variation |
| rs6866116 | 5 | 123980872 | T>C | 4 | 68 | / | Yes | / | 2+ | Non-coding variation |
| rs79679768 | 5 | 124034164 | A>G | 4 | 150 | 5 | No | / | / | Non-coding variation |
| rs10042766 | 5 | 123989154 | T>C | 3 | 303 | / | Yes | / | 1+ | Non-coding variation |

Chr: chromosome. Allele: ref allele > alt allele. Score: the score of the variation from VARAdb. N_enhancer: The number of enhancers and super enhancers the variation locates in. N_ATAC: The number of accessible chromatin regions the variation locates in from TCGA and Cistrome. Risk SNP: The variation is a risk SNP. N_eQTL: The number of eQTL pairs the variation owns from GTEx v7, PancanQTL and HaploReg v4.1. N_disease: The diseases or traits or phenotypes are associated with the variation. These diseases/traits/phenotypes were integrated from DisGeNet 6.0, GWAS relevant information and cis-eQTL data of 33 cancers from TCGA. Variation_type: Coding region variation or non-coding variation.

**Table S5.** Functional annotation of five SNPs in *ZNF608* using 3DSNP v2.0.

| **Variant ID** | **Ref/Alt** | **Score** | **3D interacting gene** | **Enhancer** | **Promoter** | **TFBS** | **Motif** |
| --- | --- | --- | --- | --- | --- | --- | --- |
| rs10067299 | G/A | 94.39 | *ZNF608* | 83 | 5 | 15 | 1 |
| rs79679768 | A/G | 56.51 | *ZNF608* | 62 | 6 | 1 |  |
| rs10042766 | T/C | 32.25 | *ZNF608* | 44 |  |  |  |
| rs6862252 | T/G | 8.07 | *ZNF608* | 17 |  |  |  |
| rs6866116 | T/C | 5.67 | *ZNF608* | 12 |  |  |  |

Ref: reference allele. Alt: alternative allele. TFBS: transcription factor binding sites.

**Table S6**. Description of top ten gene sets (related to Fig. 4F).

| **Standard name** | **Brief description** | **Detailed description** |
| --- | --- | --- |
| GSE39110_UNTREATED_VS_IL2_TREATED_CD8_TCELL_DAY6_POST_IMMUNIZATION_DN | Genes down-regulated in CD8 T cells 6 days after immunization: control versus IL2 treatment. | Genes involved in amplified CD8^+^ T memory development induced by a homologous prime-boost immunization protocol with transiently enhanced IL-2R signaling. |
| GSE2706_R848_VS_R848_AND_LPS_2H_STIM_DC_UP | Genes up-regulated in comparison of dendritic cells (DC) stimulated with R848 at 2 h versus DCs stimulated with LPS (TLR4 agonist) and R848 for 2 h. | Genes are responsible for initiating adaptive immune responses by activation of dendritic cells (DCs) induced by toll-like receptors (TLRs). LPS and the synthetic imidazoquinoline resiquimod (R848) trigger TLR4 and TLR8, respectively. |
| GSE39022_LN_VS_SPLEEN_DC_DN | Genes down-regulated in dendritic cells from: lymph node versus spleen. | Genes responsible for differential capacity of inducing and retaining iTreg cells in dendritic cells derived from spleen and lymph node. |
| GSE36009_UNSTIM_VS_LPS_STIM_DC_UP | Genes up-regulated in dendritic cells: control versus LPS. | Genes potentially involved in Nlrp10-dependent DC function on Nlrp10-deficient BMDCs treated with or without LPS. |
| GSE21774_CD62L_POS_CD56_DIM_VS_CD62L_NEG_CD56_DIM_NK_CELL_UP | Genes up-regulated in SELL dim NK cells: NCAM1+ versus NCAM1-. | Genes involved in the heterogeneity of CD56^dim^ cells identified according to the expression of *CD26L*. |
| GSE30083_SP2_VS_SP4_THYMOCYTE_UP | Genes up-regulated in comparison of SP2 thymocytes versus SP4 thymocytes. | Genes involved in the global program of gene expression during the maturation of murine CD4 single positive thymocytes. |
| GSE5589_LPS_AND_IL10_VS_LPS_AND_IL6_STIM_MACROPHAGE_45MIN_DN | Genes down-regulated in bone marrow-derived macrophages (45 min): IL10 and LPS versus IL6 and LPS. | Gene expression changes in response to IL-6 or IL-10 stimulation in the presence of lipopolysaccharide. |
| GSE6674_CPG_VS_CPG_AND_ANTI_IGM_STIM_BCELL_DN | Genes down-regulated in B lymphocytes: CpG oligodeoxynucleotide 1826 versus anti IgM and CpG oligodeoxynucleotide 1826. | Genes involved in autoreactive B cells, stimulated by a combination of BCR and TLR9 ligands, including IL-2, which may contribute to the activation of additional cells involved in the autoimmune disease process. |
| GSE40274_LEF1_VS_FOXP3_AND_LEF1_TRANSDUCED_ACTIVATED_CD4_TCELL_UP | Genes up-regulated in CD4 T conv over-expressing LEF1 versus LEF1 and FOX3. | Genes involved in Lef1, along with other transcription factors, synergizes with FoxP3 to activate the Treg transcriptional signature and enhance FoxP3 occupancy at its genomic targets. |
| GSE13522_CTRL_VS_T_CRUZI_G_STRAIN_INF_SKIN_UP | Genes up-regulated in skin from BALB/c mice after injection of: control versus Trypanosoma cruzi (strain G). | Genes involved in the early host response triggered G strain of Trypanosoma cruzi at a local infection sitevin a murine intradermal infection model. |

**Fig S1. The distribution of weighted polygenic risk score (wPRS) based on 5 SNPs in *ZNF608*.** (A) Density plot and (B) Box plot of wPRS between cases with allergic rhinitis and control.


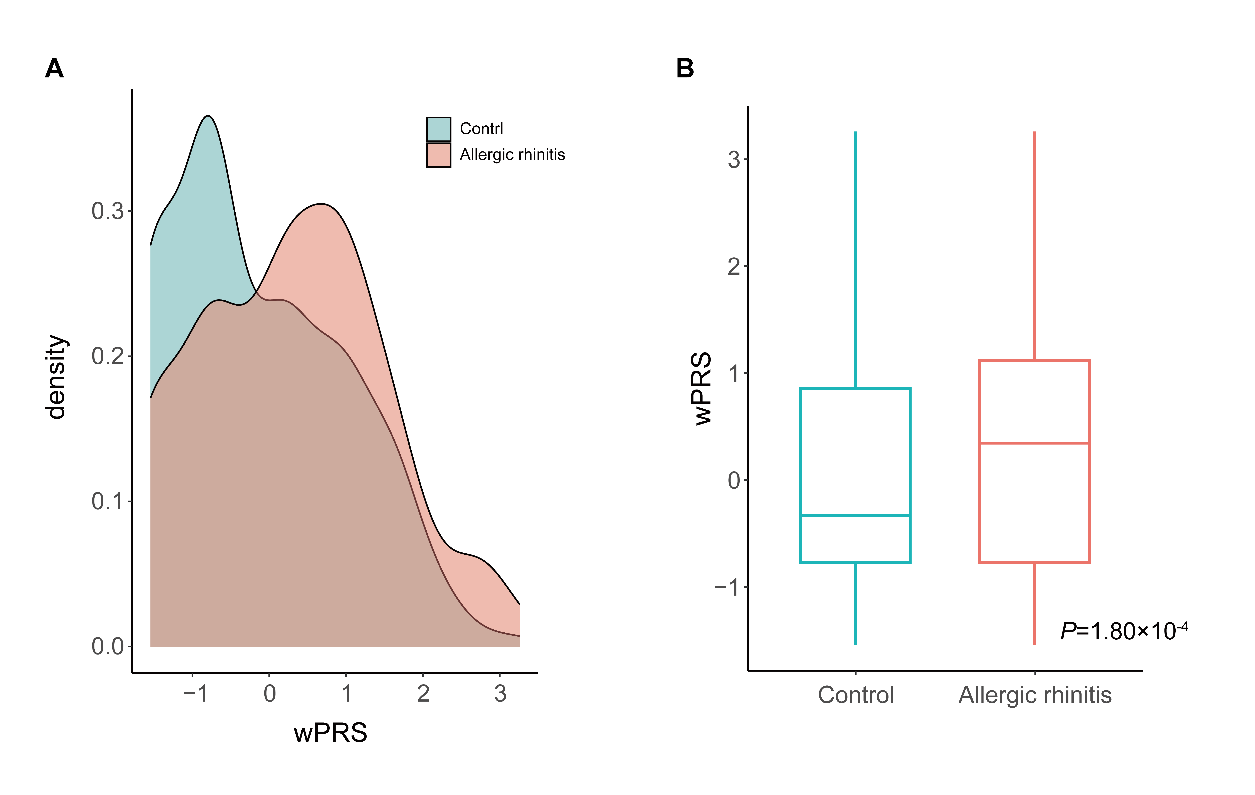
**Fig S2.** **Visualization of chromatin loops, states and 3D interaction of SNPs in *ZNF608* using 3DSNP.** (A-C) Circos plots of rs6862252, rs6866116, and rs10042766.


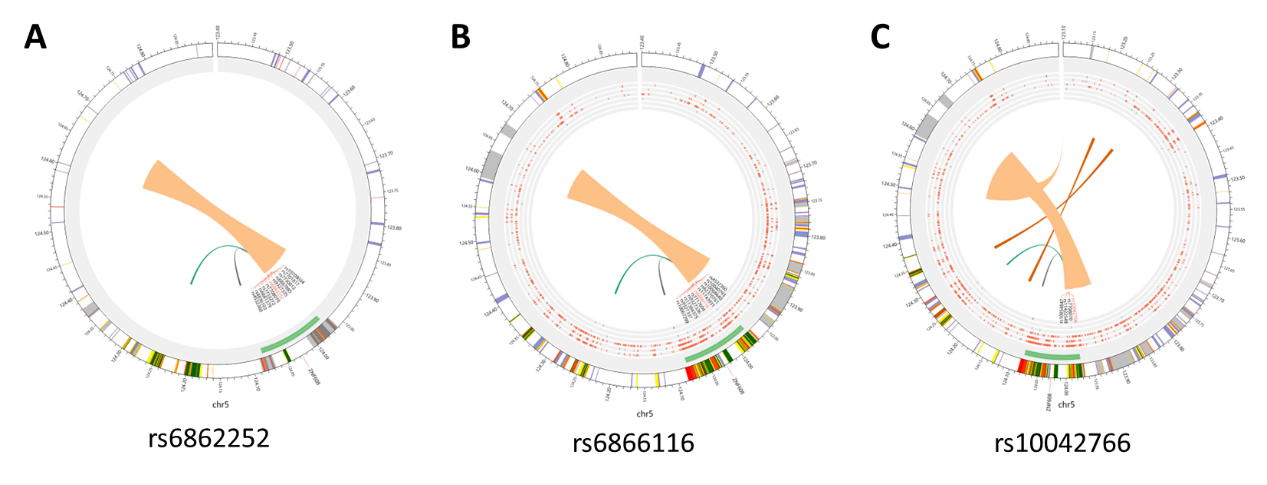


**Fig S3. The change of RNA secondary structure and minimum free energy (MFE) caused by rs6862252 T>G and rs10042766 C>T based on RNAfold website**. (A-B) Mountain plots present the MFE structure, the thermodynamic ensemble of RNA structures, and the centroid structure of rs6862252 and rs10042766, as well as the positional entropy for each position. Plots were obtained from RNAfold web server.


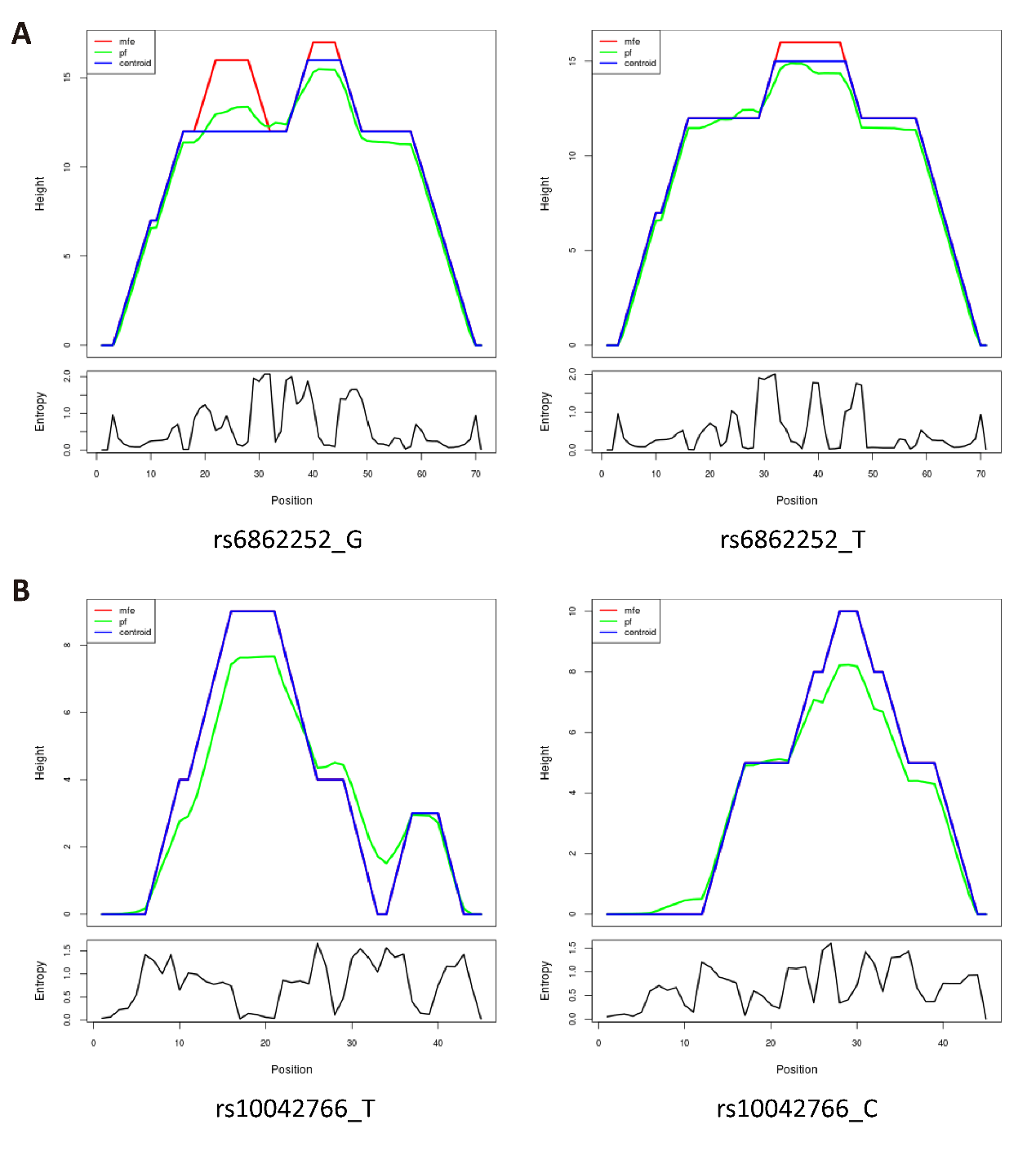

Supplement: Supplementary file 1 — Supporting Information S1 [file CLT2-15-e70081-s001.docx]
